# Effect of COVID-19 Precautions on Neonatal Resuscitation Practice: A Balance Between Healthcare Provider Safety, Infection Control, and Effective Neonatal Care

**DOI:** 10.3389/fped.2020.00478

**Published:** 2020-08-18

**Authors:** Brenda Hiu Yan Law, Po-Yin Cheung, Khalid Aziz, Georg M. Schmölzer

**Affiliations:** ^1^Centre for the Studies of Asphyxia and Resuscitation, Royal Alexandra Hospital, Edmonton, AB, Canada; ^2^Department of Pediatrics, University of Alberta, Edmonton, AB, Canada

**Keywords:** infants, newborn, delivery room, neonatal resuscitation, COVID-19

## Abstract

Adaptations have been proposed for resuscitation of infants born to women with COVID-19, to protect health care providers, maintain infection control, and limit post-natal transmission. Changes especially impact respiratory procedures, personal protective equipment (PPE) use, resuscitation environments, teamwork, and family involvement. Adding viral filters to ventilation devices and modifications to intubation procedures might hinder effective ventilation. PPE could delay resuscitation, hinder task performance, and degrade communication. Changes to resuscitation locations and team composition alter workflow and teamwork. Physical distancing measures and PPE impede family-integrated care. These disruptions need to be considered given the uncertainty of vertical transmission of SARS-CoV-2.

## Introduction

COVID-19 is an evolving pandemic caused by the novel betacoronavirus SARS-CoV-2 (1). While mortality is associated with advanced age and co-morbidities, pregnant women can be affected (2). It is unclear how much maternal COVID-19 infection contributes to fetal distress, preterm labor, or indications for early delivery (3). Suspected vertical transmission has been reported (4) but the risk appears low (5).

Current neonatal resuscitation guidelines include recommendations on equipment, procedures, team composition, and teamwork (6). Adaptations have been proposed for resuscitation of infants born to women with suspected or confirmed COVID-19, to protect health care providers (HCPs), limit post-natal transmission, and maintain infection control (7). Neonatal resuscitation may be especially impacted by changes in (i) respiratory support, (ii) personal protective equipment (PPE), (iii) resuscitation environment, (iv) team-based activities, and (v) family involvement (Table 1). In this article, we explore these potential disruptions and propose strategies to evaluate and minimize their impact.

**Table 1:** Examples of changes in routine neonatal resuscitation practices that may disrupt care.

**Change Domain**	**Examples**	**Potential disruption**
Respiratory support: ventilation equipment and Procedures	Viral Filters	Viral filters on mask ventilation devices introduce resistance, dead space and potential for obstruction
	Endotracheal Intubation	Uncertainty with regards to timing and indication of endotracheal intubations may cause deviations from standard practice
	Respiratory Support Devices	Altering respiratory support to minimize AGPs increases HCP workload and might subject neonates to interventions not best suited to their respiratory needs
Personal protective equipment: physical and time constraints	Time to Don PPE	Time to don PPE might delay resuscitation
	Effect on Performance	PPE might affect HCP vision, increase discomfort, and heighten anxiety
	Effect on Communication	N95 masks might decrease speech intelligibility
Physical environment: location, layout and transfer	Location of Delivery Rooms	Use of dedicated COVID-19 delivery rooms and operating rooms farther away from the NICU increases neonatal team travel time and may cause confusion
	Not Using Neonatal Resuscitation Room	Performing resuscitations in the delivery room, instead specialized neonatal resuscitation room, takes away organized resuscitation environment, and disrupts flow of post-natal management
	Limited Equipment in the room	Non-essential equipment to be kept outside of the delivery/operating room to avoid contamination, decreasing access to equipment such as organized emergency equipment cart
		COVID-19 resuscitation pack replacing emergency equipment cart, and is less organized and new to HCPs
	Transport to NICU	To reduce contamination, infants transported in incubator with separate transport team, increasing HCP workload, and introducing delays
Team-based activities: team preparedness, structure, and communication	Limit Team Members in the Room	Minimal team members changes roles, increasing individual workload, introduces delays for procedures
	Communication Within Neonatal Team	Team members awaiting outside of the room introduces communication barriers
	Communication Between Teams	Need to communicate between the anesthetic, obstetric, and neonatal teams to share information regarding COVID-19 status and when alert when AGPs are performed on mother and/or neonate
	Exclude Trainees	Decrease learning opportunities and affect future competence in independent practice
Effects on Family-Integrated care	Communication and Support	Move to electronic platforms can be a barrier to communication to family in antenatal and postnatal care
		Absence of family during resuscitation may hinder communication and shared decision-making
	Parental-Infant Bonding	Parental separation, masking may affect bonding

## Ventilation Equipment and Procedures

Mask ventilation and endotracheal intubation are aerosol generating procedures (AGP) critical to neonatal resuscitation (6). The risk of transmitting SARS-CoV-2 to HCPs during AGPs is estimated from observational studies in other viral infections (8). Modifications to ventilation practices during neonatal resuscitation have been proposed to protect HCPs during AGPs, based on limited evidence on vertical transmission and aerosolization of SARS-CoV-2 (7, 9).

General COVID-19 resuscitation guidelines recommend the use of viral filters on mask ventilation devices to decrease risks to HCPs (9). The effectiveness of viral filters depend on mask seal, filter, and device type (e.g., self-inflating bag, flow-inflating bag, and T-piece resuscitators), flow rate, filter integrity, and patient factors (10). Experience with filters come from ventilated patients in intensive care or anesthetic environments (11) Filters increase airway resistance, dead space and CO_2_ retention, and obstruct the ventilation circuit if soiled (10, 11). Filter complications may be exaggerated in neonates due to their lower tidal volume, minute ventilation, and functional reserve (11). Smallest filters are only rated for infants weighing 3 kg and above. Manufacturers have warned against fitting of viral filters to their devices, as these configurations are untested (12). Further testing is needed to determine how filters affect mask ventilation in neonates. Another strategy to reduce aerosolization during mask ventilation is to minimize mask leak. Two-person mask application reduces mask leak but increases HCP exposure; respiratory function monitoring detects mask leak but requires additional training and equipment (13).

Endotracheal intubation is a confirmed risk factor for HCP infection in the previous SARS (Severe Acute Respiratory Syndrome) outbreak (14). Given the low likelihood of vertical transmission with the SARS-CoV-2, the need for COVID-19 intubation precautions during neonatal resuscitation in the delivery room is likely low. Pre-emptive intubation to avoid mask ventilation (as suggested in adult settings) may lead to excess intubations and intubation attempts in neonates. The use of videolaryngoscopes and cuffed endotracheal tubes further minimize risk during neonatal resuscitation, but are significant practice changes for many centers. At our center, we have not altered the indications, timing, or procedure for intubation during neonatal resuscitation of COVID-19 exposed infants. Nonetheless, concerns with this procedure may cause deviations from established practice.

Changes to respiratory equipment or indications for their use may affect HCP workload, introduce equipment uncommon in neonatal care, or lead to neonates receiving supports or interventions unsuited to their needs. Examples include: using low flow nasal canula (LFNC) instead of nasal CPAP (constant positive airway pressure) to avoid AGPs, intubating with cuffed endotracheal tubes, or inserting viral filters in respiratory circuits. The impact of these approaches may be unpredictable. If vertical transmission is unlikely (5), the potential harms of these variations may outweigh potential benefits, particularly if HCPs don PPE.

## Personal Protection Equipment

HCP roles and tasks are variably impacted by personal protective equipment (PPE) (15). Although opinions vary with respect to use of respirator (N95) vs. surgical masks for neonatal resuscitation (7, 16), both may impact HCP performance. Guidelines support use of PPE in suspected or confirm COVID-19 settings, even if initial resuscitation is delayed (9). Donning PPE introduce delays when neonatal resuscitation is not anticipated, and may be done incorrectly if HCPs are rushed. When the likelihood of resuscitation is low, HCP exposure, and PPE use may be minimized by limiting the resuscitation team to 1 or 2 members. This approach acknowledges that advanced resuscitation (e.g., umbilical venous line insertion, epinephrine), if required, may be delayed. Some advocate that possible delays to emergency c-sections should be discussed with pregnant women under COVID-19 isolation (17). Similar discussions for neonatal resuscitation may be needed.

Donning and doffing PPE take practice, time, and care for full effect. When pressed for time, HCPs may don and doff incorrectly (18). Additional staff may be engaged to “spot” breaches and prevent self-contamination during doffing. PPE itself may further impact individual performance (19). The effect of masks and eye-protection on performance of neonatal resuscitation tasks is unknown. Eye-protection or fogging may hinder vision, interfering with clinical assessments and procedures such as intubation. Respirator masks create resistance to breathing and can cause anxiety and discomfort.

Finally, PPE might disrupt interpersonal communication. Face coverings can obscure both verbal and non-verbal communication. Masks can decrease speech intelligibility (20), particularly in a noisy resuscitation environment. Speech can be further distorted through devices (e.g., speaker phones) used to communicate with HCPs outside of the resuscitation room. Simulation training can help ensure safe use and familiarity with PPE, but practice with PPE can be limited by the need to conserve supplies.

## Resuscitation Environment

In addition to PPE, HCPs experience modifications to the physical resuscitation environment. Specific labor and operating rooms may be designated for women requiring COVID-19 precautions (7). Negative pressure isolation rooms are preferred for ongoing care, if available. Not uncommonly, designated rooms are remote from the neonatal unit, not designed for isolation, and previously reserved for cases not needing neonatal equipment or team involvement. Room changes can increase travel time for the neonatal team and may delay responses due to unfamiliarity with room location and layout.

For COVID-19 cases, there are conflicting opinions on whether neonatal resuscitation should occur directly in the delivery room (DR) (7, 16). Remaining in the DR reduces the extent of contamination. On the other hand, resuscitation in a space separate from the mother (i) limits exposure of obstetrical HCPs to neonatal AGP, (ii) removes the neonate and the neonatal team from exposure to maternal infection, and (iii) allows for a dedicated resuscitation space. Some centers have rooms adjacent to DR and operating rooms for neonatal resuscitation and stabilization, ergonomically organized with equipment, and sterile supplies. These areas are infrequently designed for isolation or decontamination. Resuscitation in the DR changes the access to such organized equipment and alters workflow. Accessibility to equipment may be improved using modular, portable emergency packs that contain basic neonatal resuscitation equipment. Non-urgent interventions (e.g., line insertion), may be deferred in favor of transport to the Neonatal Intensive Care Unit (NICU) for procedures. Subsequent downstream disruptions include increased NICU workload and potential delays in procedures which would have previously occurred before transfer to the NICU. Conversely, for units used to resuscitations in the DR, resuscitating in a separate room can also be disruptive.

When transporting a potentially infected neonate to the neonatal unit, avoidance of environmental contamination and bystander exposure are priorities (21). Transporting a neonate in an enclosed incubator with a clean transport team in PPE may minimize contamination but requires additional personnel, PPE consumption, and choreographing of patient movement. Enclosed incubators are not airtight and may emit generated aerosols, contaminating hospital corridors, and putting bystanders at risk. Additional precautions include placing viral filters on ventilators to filter expired gases. The need for careful neonatal transport may (i) increase HCP workload, (ii) delay further interventions (e.g., venous access and intubation for surfactant), and (iii) increase the risk for hypothermia in small infants. Possible solutions include dedicated or escorted routes through the facility that are free of visitors and non-essential staff. Drills or briefings that clarify the physical environment, equipment, and transfer route will mitigate some of these risks.

## Team Structure and Communications

Neonatal resuscitation team size and composition vary. For hospitals used to larger teams for complex resuscitations, decreasing team size to limit HCP exposure may disrupt team function. Some potential changes include: (i) team leader performing procedures such as airway management, (ii) recorder staying outside the resuscitation room, and (iii) additional HCPs and trainees waiting outside the room. During *in-situ* simulations, we encountered communication breakdowns between those inside and outside the resuscitation room causing inaccuracies in timing chest compressions, incomplete resuscitation records, and delays in activating additional personnel. Communication failures can further cause errors through failures in shared situational awareness and team coordination.

Neonatal, obstetrical, and anesthetic teams need to share COVID-19-related information during briefings and timeouts, and in real-time. This should help the neonatal team prepare for COVID-19 precautions, as well as minimize personnel in the operating room if a mother is being intubated and extubated. By alerting when AGP are performed, neonatal HCPs can give obstetrical team members the opportunity to take suitable precautions. These communication practices will need training and reinforcement to ensure that they occur consistently.

Finally, HCP trainees may be excluded from resuscitations of infants born to women under COVID-19 isolation. In Canada, pediatric residents report a low level of confidence in their neonatal clinical skills and may only have limited exposure to neonatal resuscitation in their training (22). If COVID-19 or other similar communicable diseases become prevalent, this approach will negatively impact our ability to train HCPs. Strategies are needed that facilitate the training of both established team members and trainees in the “new normal” of this and future pandemics.

## Family and Other Impacts

Policies for increased physical distancing and isolation are highly disruptive to family-integrated care. For example, while telehealth can facilitate physical distancing for antenatal consultations, effective electronic communication can be challenging for sensitive conversations surrounding resuscitation and goals of care. Limiting family presence during active neonatal resuscitation can further impact communication, availability of emotional support, and shared decision-making. Infection control practices, such as face masks or isolation, are barriers to mother-infant interaction and bonding. Opinions on mother-infant contact post-partum differ with a balance of benefits (e.g. bonding, establishing breastfeeding, and aiding postnatal transition) against concerns for postnatal COVID-19 transmission (7, 9, 16). Involvement of parent advocates in policy development can help sustain efforts to provide safe and compassionate care during this pandemic.

**Figure 1:**
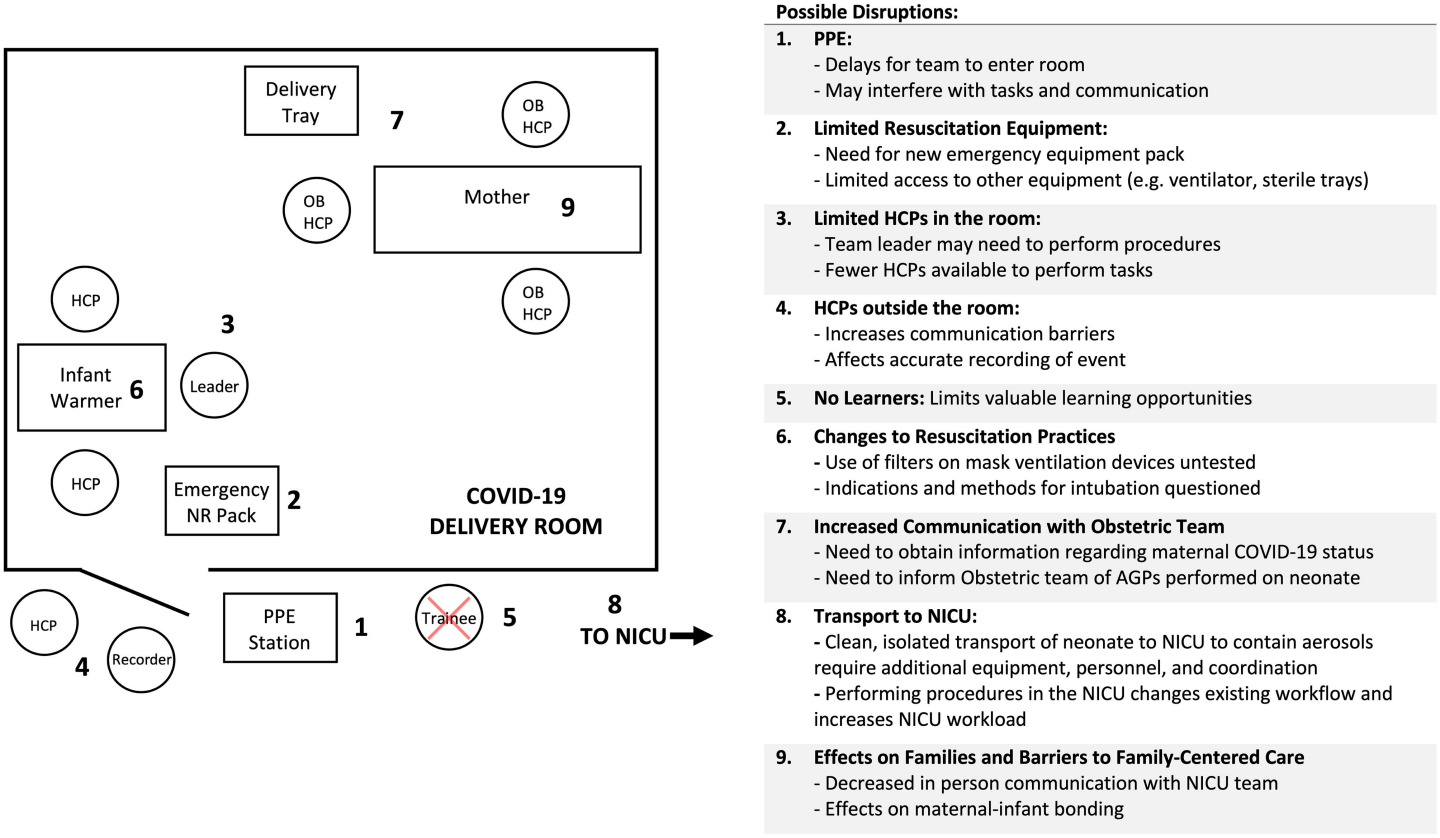
High risk COVID-19 delivery room layout and changes to neonatal resuscitation workflow (HCP, Healthcare Provider; OB, Obstetrics; PPE, Personal Protective Equipment; NR, Neonatal Resuscitation; NICU, Neonatal Intensive Care Unit).

Together, these potential impacts are illustrated in Figure 1.

## Strategies to Examine and Minimize the Impact of Covid-19 Practice Changes on Neonatal Resuscitation

Probably the most effective path to maximizing patient and HCP safety during neonatal resuscitation is via efficiently identifying pregnant women with suspected COVID-19 prior to delivery. Over- or under-identification represent risk to the woman, the baby and/or the clinical team.

Clear rationales for practice changes as well as staff familiarity with procedure and equipment modifications will ensure that both risks and disruptions are minimized. Tabletop exercises, *in-situ* walkthroughs, and real-time simulations can help achieve these goals, aiding protocol development, testing, and knowledge dissemination (23). Simulations can involve just the neonatal team, or include obstetrical and anesthetic members. Local infection prevention and control (IPC) involvement ensures IPC standards are met. Frequent communication between protocol development and resuscitation teams keep frontline HCPs up to date; HCPs have identified clear communication, training, and support from managers as facilitators to following IPC guidelines (24). Ideally, frontline HCPs are involved from the planning stages to detect and mitigate impact on workflow. Continuous evaluation ensures that plans live up to the challenge of reality. At our center, teams are encouraged to provide feedback with each COVID-19 encounter. During initial stages, medical and IPC leads were notified of each COVID-19 encounter and actively solicited *post-hoc* feedback from frontline HCPs. Successes and challenges were shared, and protocols amended or clarified as needed. Multidisciplinary representation in our local COVID-19 response team and a dedicated neonatal resuscitation team nurse coordinator provided multiple mechanisms for HCP to provide feedback. As our frontline HCPs gained confidence and comfort in our protocols, daily in-person team huddles, and email communication facilitated ongoing feedback, communication, and support. Successful communication strategies will vary depending on unit culture, size, and acuity. Information sharing between NICUs in our region helped further refine protocols as successful strategies and solutions were rapidly disseminated.

As we learn more about perinatal transmission of COVID-19, protocols should be amended to minimize disruptions. Future redesign of neonatal resuscitation equipment, spaces, training, and teams could better address infection control and protection. Technologies such as smart glasses can provide real-time visual-audio links between team members in different rooms to help maintain team communication.

## Conclusion

When resuscitating infants born to women with suspected or confirmed COVID-19 infection, adaptations to current guidelines are considered to protect HCPs and maintain infection control. Adaptations involve changes to ventilation procedures and equipment, use of PPE, decreasing team size, and physical distancing measures. These adaptations are assisted by early identification of maternal COVID-19 status, real-time simulations, continuous protocol evaluation, and team communication strategies. Although safety for all involved remains a priority, these changes can be disruptive and should be re-evaluated as more information emerges around the perinatal transmission of this SARS-CoV-2.

## Data Availability Statement

The original contributions presented in the study are included in the article/supplementary material, further inquiries can be directed to the corresponding author/s.

## Author Contributions

Conception and design: BL, P-YC, KA, and GS. Drafting of the article: BL, P-YC, KA, and GS. Critical revision of the article for important intellectual content: BL, P-YC, KA, and GS. Final approval of the article: BL, P-YC, KA, and GS. All authors contributed to the article and approved the submitted version.

## Conflict of Interest

The authors declare that the research was conducted in the absence of any commercial or financial relationships that could be construed as a potential conflict of interest.

## References

[1] Shah SGS Farrow A. A commentary on “World Health Organization declares global emergency: A review of the 2019 novel Coronavirus (COVID-19)”. Int J Surg. (2020) 76:128–29. 10.1016/j.ijsu.2020.03.001 32169574 PMC7128929

[2] Chen H Guo J Wang C Luo F Yu X Zhang W. Clinical characteristics and intrauterine vertical transmission potential of COVID-19 infection in nine pregnant women: a retrospective review of medical records. Lancet. (2020) 395:809–15. 10.1016/S0140-6736(20)30360-3 32151335 PMC7159281

[3] Mullins E Evans D Viner RM O'Brien P Morris E. Coronavirus in pregnancy and delivery: rapid review. Ultrasound Obstet Gynecol. (2020) 55:586–92. 10.1002/uog.22014 32180292

[4] Kirtsman M Diambomba Y Poutanen SM Malinowski AK Vlachodimitropoulou E Parks WT. Probable congenital SARS-CoV-2 infection in a neonate born to a woman with active SARS-CoV-2 infection. CMAJ. (2020) 192:E647–50. 10.1503/cmaj.200821 32409520 PMC7828840

[5] Schwartz DA. An analysis of 38 pregnant women with COVID-19, their newborn infants, and maternal-fetal transmission of SARS-CoV-2: maternal coronavirus infections and pregnancy outcomes. Arch Pathol Lab Med. (2020) 144:799–805. 10.5858/arpa.2020-0901-SA 32180426

[6] Wyckoff MH Aziz K Escobedo MB Kapadia VS Kattwinkel J Perlman JM. Part 13: neonatal resuscitation: 2015 American Heart Association guidelines update for cardiopulmonary resuscitation and emergency cardiovascular care. Circulation. (2015) 132(18 Suppl 2):S543–6. 10.1161/CIR.0000000000000267 26473001

[7] Chandrasekharan P Vento M Trevisanuto D Partridge E Underwood MA Wiedeman J Neonatal resuscitation and postresuscitation care of infants born to mothers with suspected or confirmed SARS-CoV-2 infection. Am J Perinatol. (2020) 37:813–24. 10.1055/s-0040-1709688 32268381 PMC7356083

[8] Tran K Cimon K Severn M Pessoa-Silva CL Conly J. Aerosol generating procedures and risk of transmission of acute respiratory infections to healthcare workers: a systematic review. PLoS One. (2012) 7:e35797. 10.1371/journal.pone.0035797 22563403 PMC3338532

[9] Edelson DP Sasson C Chan PS Atkins DL Aziz K Becker LB. Interim guidance for basic and advanced life support in adults, children, and neonates with suspected or confirmed COVID-19: from the emergency cardiovascular care committee and get with the guidelines[(R)]-resuscitation adult and pediatric task forces of the American Heart Association in Collaboration with the American Academy of Pediatrics, American Association for Respiratory Care, American College of Emergency Physicians, The Society of Critical Care Anesthesiologists, and American Society of Anesthesiologists: Supporting Organizations: American Association of Critical Care Nurses and National EMS Physicians. Circulation. (2020) 141:e933–e43. 10.1161/CIRCULATIONAHA.120.047463 32270695 PMC7302067

[10] Wilkes AR. Heat and moisture exchangers and breathing system filters: their use in anaesthesia and intensive care. Part 2 - practical use, including problems, and their use with pediatric patients. Anaesthesia. (2011) 66:40–51. 10.1111/j.1365-2044.2010.06564.x 21118189

[11] Whitelock DE de Beer DA. The use of filters with small infants. Respir Care Clin N Am. (2006) 12:307–20. 10.1016/j.rcc.2006.03.006 16828697

[12] Fisher and Paykel Healthcare COVID-19 Resource Center. Available online at: https://www.fphcare.com/en-ca/covid-19/?fbclid=IwAR1ED1ss-fXARbS8tXStT1D5643Cp9vfvXmBT5v53nTClgYFH8A3Ggvw3x (cited April 20, 2020).

[13] Tracy MB Klimek J Coughtrey H Shingde V Ponnampalam G Hinder M. Mask leak in one-person mask ventilation compared to two-person in newborn infant manikin study. Arch Dis Child Fetal Neonatal Ed. (2011) 96:F195–200. 10.1136/adc.2009.169847 21071683

[14] Fowler RA Guest CB Lapinsky SE Sibbald WJ Louie M Tang P. Transmission of severe acute respiratory syndrome during intubation and mechanical ventilation. Am J Respir Crit Care Med. (2004) 169:1198–202. 10.1164/rccm.200305-715OC 14990393

[15] Harrod M Petersen L Weston LE Gregory L Mayer J Samore MH. Understanding workflow and personal protective equipment challenges across different healthcare personnel roles. Clin Infect Dis. (2019) 69(Suppl 3):S185–S91. 10.1093/cid/ciz527 31517971

[16] Narvey M Delivery room considerations for infants born to mothers with suspected or proven COVID-19. Canadian Pediatric Society. Available online at: https://www.cps.ca/en/documents/position/delivery-room-considerations-infants-born-to-mothers-with-suspected-or-proven-covid-19 (cited 2020 May 6).

[17] Morris E O'Brien P Goodyear G Relph S Jardine J Powell A Coronavirus (COVID-19) Infection in Pregnancy: Information for Healthcare Professionals. Royal College of Obstetricians and Gynecologists (UK). Available online at: https://www.rcog.org.uk/globalassets/documents/guidelines/2020-04-03-coronavirus-covid-19-infection-in-pregnancy.pdf (cited April 20, 2020).

[18] Mulvey D Mayer J Visnovsky L Samore M Drews F. Frequent and unexpected deviations from personal protective equipment guidelines increase contamination risks. Am J Infect Control. (2019) 47:1146–7. 10.1016/j.ajic.2019.03.013 31027940

[19] Al-Ghamri AA Murray SL Samaranayake VA. The effects of wearing respirators on human fine motor, visual, and cognitive performance. Ergonomics. (2013) 56:791–802. 10.1080/00140139.2013.767383 23514088

[20] Radonovich LJ Jr Yanke R Cheng J Bender B. Diminished speech intelligibility associated with certain types of respirators worn by healthcare workers. J Occup Environ Hyg. (2010) 7:63–70. 10.1080/15459620903404803 19904661

[21] Ong SWX Tan YK Chia PY Lee TH Ng OT Wong MSY. Air, surface environmental, and personal protective equipment contamination by severe acute respiratory syndrome coronavirus 2 (SARS-CoV-2) from a symptomatic patient. JAMA. (2020) 323:1610–2. 10.1001/jama.2020.3227 32129805 PMC7057172

[22] Cormier S Chan M Yaskina M van Manen M. Exploring pediatric residents' perceptions of competency in neonatal intensive care. Paediatr Child Health. (2019) 24:25–9. 10.1093/pch/pxy061 30792597 PMC6376347

[23] World Health Organization WHO Simulation Exercise Manual. 2017 WHO-WHE-CPI-2017.10 (2017). Available online at: https://www.who.int/ihr/publications/WHO-WHE-CPI-2017.10/en/ (cited April 20, 2020).

[24] Houghton C Meskell P Delaney H Smalle M Glenton C Booth A. Barriers and facilitators to healthcare workers' adherence with infection prevention and control (IPC) guidelines for respiratory infectious diseases: a rapid qualitative evidence synthesis. Cochrane Database Syst Rev. (2020) 4:CD013582. 10.1002/14651858.CD013582 32315451 PMC7173761

